# Predicting Childhood Obesity Using Machine Learning: Practical Considerations

**DOI:** 10.3390/biomedinformatics2010012

**Published:** 2022-03-08

**Authors:** Erika R. Cheng, Rai Steinhardt, Zina Ben Miled

**Affiliations:** 1 Division of Children’s Health Services Research, Department of Pediatrics, Indiana University School of Medicine, Indianapolis, IN 46202, USA; 2 Purdue School of Science, Department of Computer Science, Indiana University Purdue University at Indianapolis, Indianapolis, IN 46202, USA;; 3 School of Engineering and Technology, Department of Electrical and Computer Engineering, Indiana University Purdue University at Indianapolis, Indianapolis, IN 46202, USA;; 4 Regenstrief Institute, Inc., Indianapolis, IN 46202, USA

**Keywords:** childhood obesity, BMI, machine learning, EHR

## Abstract

Previous studies demonstrate the feasibility of predicting obesity using various machine learning techniques; however, these studies do not address the limitations of these methods in real-life settings where available data for children may vary. We investigated the medical history required for machine learning models to accurately predict body mass index (BMI) during early childhood. Within a longitudinal dataset of children ages 0–4 years, we developed predictive models based on long short-term memory (LSTM), a recurrent neural network architecture, using history EHR data from 2 to 8 clinical encounters to estimate child BMI. We developed separate, sex-stratified models using 80% of the data for training and 20% for external validation. We evaluated model performance using K-fold cross-validation, mean average error (MAE), and Pearson’s correlation coefficient (R^2^). Two history encounters and a 4-month prediction yielded a high prediction error and low correlation between predicted and actual BMI (MAE of 1.60 for girls and 1.49 for boys). Model performance improved with additional history encounters; improvement was not significant beyond five history encounters. The combined model outperformed the sex-stratified models, with a MAE = 0.98 (SD 0.03) and R^2^ = 0.72. Our models show that five history encounters are sufficient to predict BMI prior to age 4 for both boys and girls. Moreover, starting from an initial dataset with more than 269 exposure variables, we were able to identify a limited set of 24 variables that can facilitate BMI prediction in early childhood. Nine of these final variables are collected once, and the remaining 15 need to be updated during each visit.

## 1. Introduction

While previously uncommon in young children, obesity is now a worldwide epidemic affecting over 40 million children under the age of 5 [1,2]. Obesity in childhood is associated with both adverse outcomes like hyperlipidemia, diabetes and hypertension [3–6], as well as with higher morbidity and mortality in adulthood [7]. The underlying causes of obesity are modifiable risk factors throughout the life course; these risk factors represent major causes of health inequalities [8]. Thus, the prevention of obesity is considered a national and global health priority [9].

Unhealthy weight gain during early childhood significantly increases the risk for obesity later in life [10,11], so the ability to identify children at a young age who carry the greatest risk for obesity could significantly improve prevention efforts [12]. Several important and potentially modifiable indicators of obesity have been identified during this timeframe, including rapid infant weight gain, poor infant sleep quality, birth weight, and maternal characteristics (e.g., current and pre-pregnancy weight, depression) [13,14]. Despite this, there has been relatively limited research into predictive modeling of childhood obesity risk, leaving many unanswered questions about how and when to intervene.

Existing research to evaluate obesity risk has predominantly employed logistic regression techniques, with limited success. The constraints of traditional regression approaches (e.g., restricting analyses to a relatively small number of predictors and assumptions of independence and linearity) have prompted others to examine non-linear interactions via machine learning [14–16]. Machine learning is increasingly recognized as useful for preventive care [17] because of its ability to characterize, adapt, learn, predict and analyze clinical data. However, one of the main challenges in employing machine learning in the clinical domain is that electronic health record (EHR) data are often incomplete and irregularly sampled (e.g., lacking regular time intervals between patient visits). In addition, height and weight, which are necessary to calculate BMI, are collected during pediatric visits in the first 2 years of life [18], but not routinely as pediatric appointments are often missed [19]. These issues hinder the performance of predictive models using EHR data. Recent techniques in deep learning and artificial neural networks address these issues and have the potential to predict health outcomes more accurately by using EHR data.

In this study, we used a longitudinal, EHR-derived dataset of children to investigate the medical history needed for a recurrent machine learning model to accurately predict BMI prior to age 4 years. Our secondary aim was to understand whether BMI prediction varies considerably between boys and girls, which would require separate BMI prediction models for each sex.

Previous studies have used machine learning techniques to develop obesity prediction models or to determine key determinants of obesity for designing intervention tools [14,20]. However, as discussed by Siddiqui et al. [20], very few of these studies analyze sex-specific prediction models, use large-scale datasets, or examine geographic/neighborhood exposure variables (e.g., access to food and opportunities for physical activity) [21,22,22–24] that might be associated with childhood obesity [25–27].

Existing models of childhood obesity risk also tend to focus on predictive variables that are routinely collected in clinical practice [28], and therefore tend to include only biological predictors and postnatal factors like infant sex and birthweight [29]. It has been suggested that one of the reasons for the intractability of childhood obesity is the failure to take into account the complexity and interconnectedness of contributing factors across the life course, ranging from the social, built, and economic environments to behavior, physiology, and epigenetics [30]. A number of childhood obesity risk factors that operate during the first 1000 days of life have been identified [13] and have special significance for obesity risk prediction. For instance, programming effects occurring during pregnancy increase children’s obesity risk. Adding this information could lead to improvements in a model’s ability to identify children at risk for obesity in early life, but EHR data typically contain information on maternal prenatal risk factors separately from risk factors during infancy and from measures of height and weight across childhood. The models presented in this study leverage data from a population-based, longitudinal database that combines data from multiple stages of the life course and thus add a valuable contribution to our understanding of obesity risk in early life.

Finally, the lack of effective interventions to reduce the risk for obesity in early life [31,32] suggests that efforts must be made to identify very young children with a high risk of developing obesity that could be specifically targeted for intervention. The methodology in the present paper employs long short-term memory (LSTM) [33] models to predict children’s BMI prior to age 4 using different lengths of history data, determined by the number of previous clinical encounters. LSTM is a recurrent neural network model that learns from an ordered sequence of events, in this case, prior clinical encounters of the patient. While several machine learning techniques could have been used, an LSTM model was selected because the history encounter constitutes a time series. In particular, the variables height and weight that are used to calculate BMI as well as the age of the child vary from one encounter to the next. LSTM models are particularly well suited for time-series applications and continue to outperform other architectures in various fields. For example, in Wang et al.’s analysis [34], LSTM outperformed RF, SVM, Naive Bayes, and Feed forward neural networks when predicting patient-reported outcomes using history responses from cancer patients. In other applications [35], LSTM models were used to predict post-operative risk for patients suffering from obesity and risk for complications after bariatric surgery.

## 2. Materials and Methods

### 2.1. Data Source

Data were extracted from the Obesity Prediction in Early Life (OPEL) database, a unique longitudinal, epidemiologic data repository that combines birth certificate, contextual-level, and health outcome data for 19,857 children born in Marion County, Indiana. We constructed the OPEL database by linking three independent data sources:

1. The Child Health Improvement through Computer Automation (CHICA) system; a computer-based pediatric primary care clinical decision support system that operated in eight pediatric primary care practices in Indianapolis between 2004–2019 [36]. The CHICA system includes data for over 47,000 patients on factors such as measured height and weight, demographics (e.g., child sex, age, race/ethnicity, Medicaid insurance status), and social determinants of health (e.g., parent health literacy, food and housing insecurity, parental depression, and infant feeding practices);
2. The IN Standard Certificate of Live Birth (i.e., ‘birth certificate’), which consists of 235 variables covering parental sociodemographic information as well as information on prenatal care, labor/delivery, and neonatal conditions and procedures. Birth certificate data were made available from the Marion County Public Health Department (MCPHD); and
3. The Social Assets and Vulnerabilities Indicators (SAVI) Project, which collects geocodes, organizes, and presents integrated data on communities in the 11-county Indianapolis metropolitan statistical area drawn from more than 30 federal, state, and local providers. All are linked to the lowest available geographic level [37]. SAVI is the nation’s largest community information system, with more than 10,000 time-series variables from 1980 to the present, including welfare, education, health, public safety, housing, demographics, locations of health facilities, health and human services, community facilities, and associated service areas.

Institutional Review Board approval to construct the OPEL database was obtained from the Indiana University School of Medicine. All data analyses for this study occurred on a restricted-access server provisioned specifically for research purposes.

### 2.2. Data Preprocessing

From the OPEL database, we identified 73,957 clinical encounters from 6614 children ages 0 to 4 years. Within this limited dataset, we performed data preprocessing to remove erroneous records, impute missing values, and encode variables into normalized features for use in our predictive model. For example, encounters where height decreased more than 2 inches from the previous encounter or with implausible recorded BMIs were categorized as input error. We also established valid ranges for the mother’s gestational weight gain and the child’s birth weight. Variables that were one-hot encoded (e.g., race of the mother or father) were converted to multi-class nominal variables. Finally, we deleted duplicative variables, administrative variables not directly relevant to the aims of our analysis, and variables without enough data to be useful.

This preprocessing yielded a list of 269 variables derived from the OPEL database that we initially considered for modeling (Appendix A). From this list, we performed feature reduction guided by existing peer-reviewed literature on early life obesity risk (e.g., [13]), expert opinion (ERC), and the results of a LASSO regression. Feature reduction also took into account noisy and sparsely populated variables.

### 2.3. Model Development

Our outcome of interest was BMI as defined by the Center for Disease Control and Prevention (CDC) guidelines [38]. We imputed missing and invalid BMIs using linear interpolation and height and weight data from previous encounters.

After preprocessing, we randomly selected an equal number of boy and girl patients, then split the dataset by patient such that 80% of our data was used for model training and 20% was used for model testing while maintaining an equal split according to patient sex. We normalized all input variables to values between −1 and 1. In the initial dataset, the girl class was the minority class.

We then developed separate long short-term memory (LSTM) [33] models to predict BMI using different lengths of history data, determined by the number of previous clinical encounters. We defined history data as either 2, 3, 5, or 8 prior encounters, and modeled our predictions of patient BMI at each encounter immediately following the set of history encounters. We modeled predictive variables as both fixed (e.g., maternal and paternal race, infant birthweight, mother’s age at birth) and varying (e.g., patient’s age, visit type, sleep quality) between encounters.

The model architecture consisted of an LSTM layer followed by a single Feed forward linear layer. The number of hidden nodes in the LSTM layer was set to half the number of input features. The Adam optimizer was used to update the weights in the model. Each model was trained using an input-output sequence with a varying number of history encounters. For example, when using five history encounters the model was trained to predict BMI at the sixth encounter.

Based on prior research demonstrating different obesity determinants for boys and girls [39], we developed three models: one for boys, one for girls, and a combined model for both. K-fold cross-validation [40] with k = 5 was used to evaluate each model and to estimate variabilities induced by the data selection. The accuracy of the models was measured using MAE and Pearson’s correlation coefficient (R^2^). We report the standard deviation of these metrics from the K-fold cross-validation.

## 3. Results

The feature reduction process resulted in a set of 24 exposure variables: 15 were derived from the CHICA dataset, 7 from the birth certificate, 1 from CHICA/birth certificate, and 1 from SAVI (Table 1).

**Figure 1.**
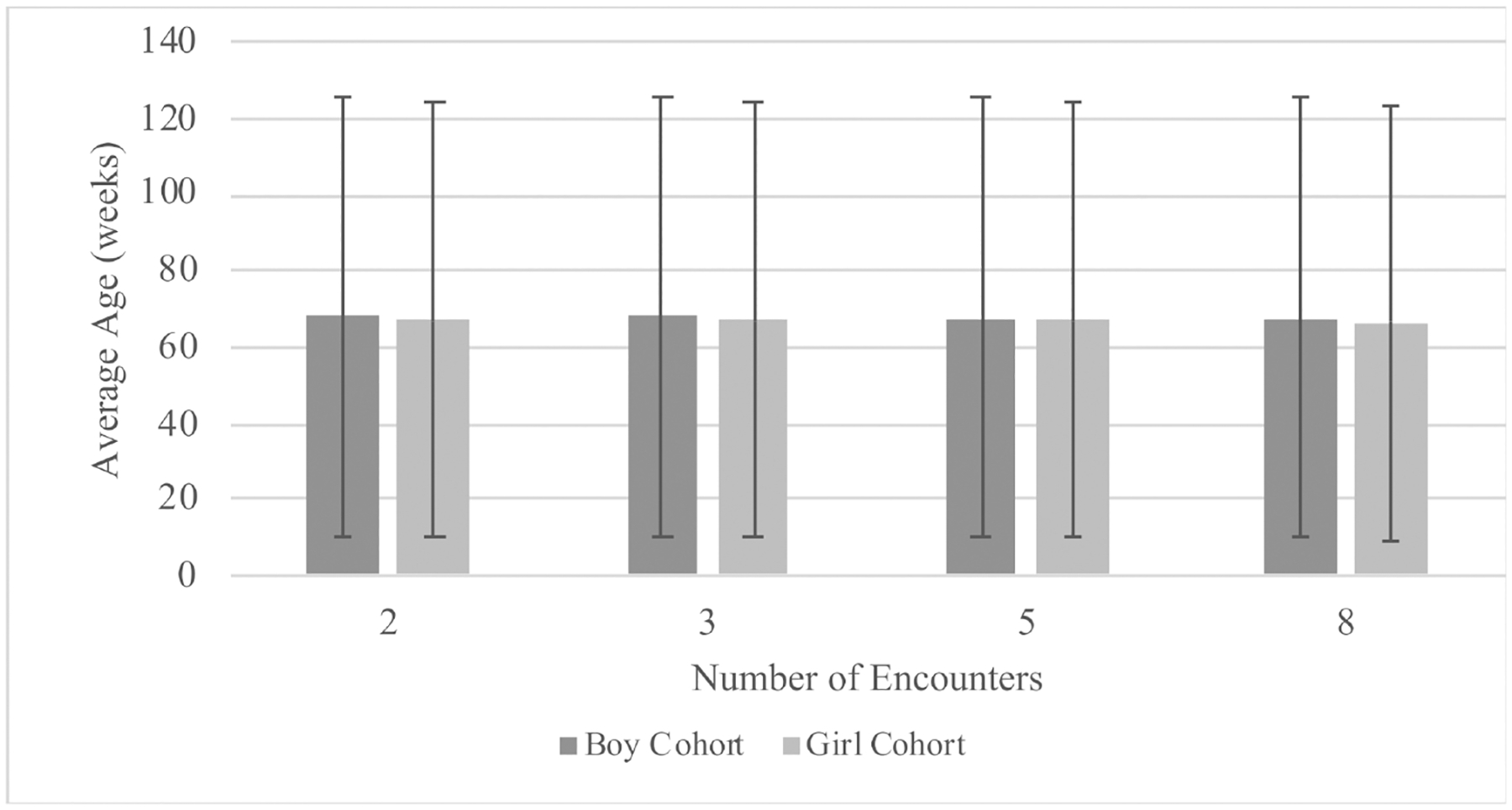
Distribution of average child age at the encounter.

Table 2 and Figure 1 show the distribution of the patients in the training and testing cohorts. As designed, there were approximately the same number of boys and girls included in both training and testing cohorts. There were no clinically meaningful differences across the cohorts in terms of mean BMI and age at the clinical encounter. The mean age at the encounter, defined as the average age across all encounters, was approximately 68 weeks (17 months), with no difference between the training and testing cohorts. There were also no significant differences between the cohorts with respect to the average number of encounters during the study period, although the average number of encounters for boys showed a higher standard deviation than for girls.

Data in Table 2 were used to develop the three types of models discussed above. The boy BMI model used a total of 2694 patients during training and was tested on 657 patients. Similarly, the girl model was trained on 2614 patients and tested on 649 patients. The combined model was trained using both training cohorts (i.e., 5308 boy and girl patients) and was tested on the combined testing cohorts (i.e., 1306 boy and girl patients).

**Figure 2.**
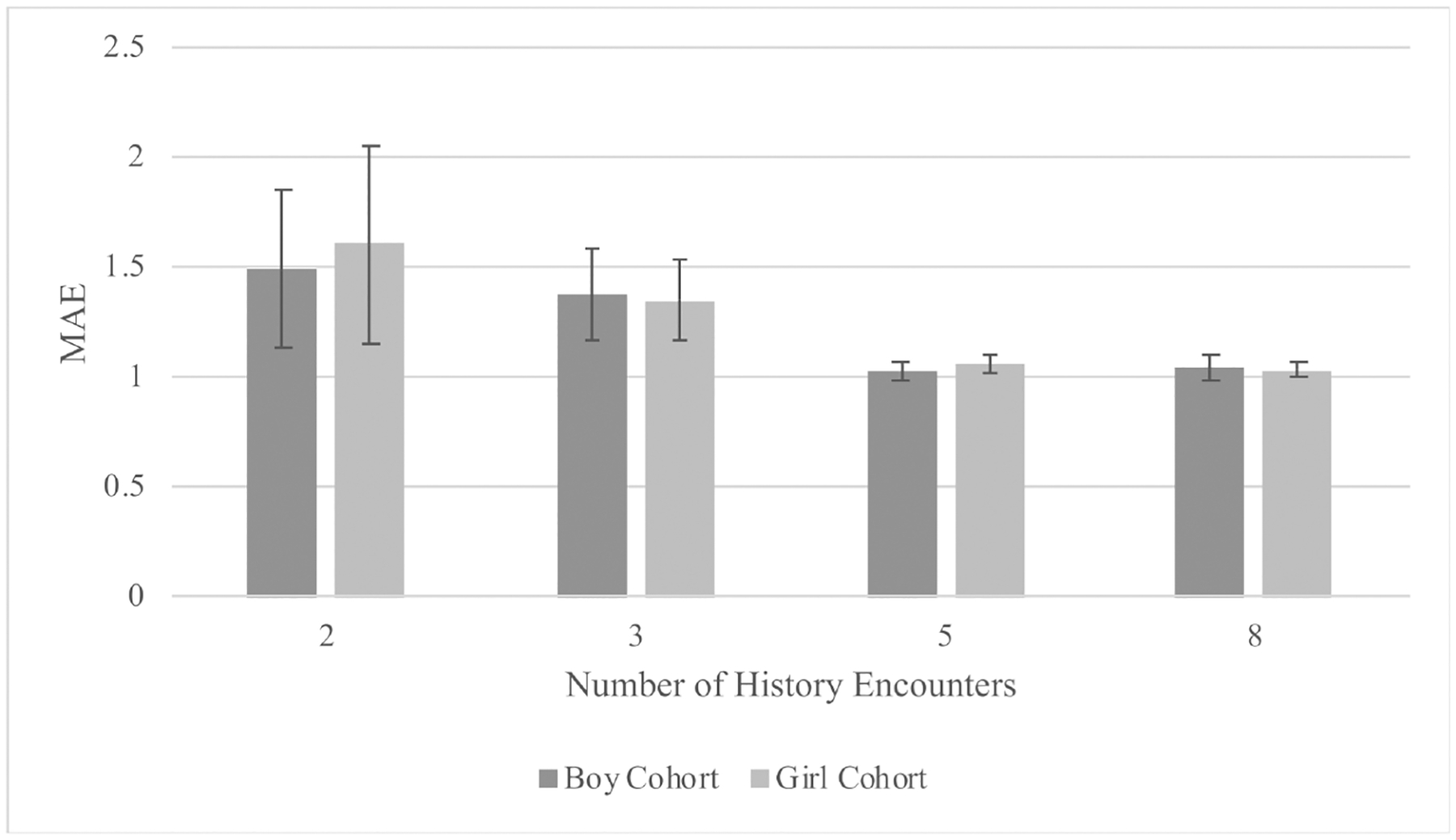
Results from the long short-term memory (LSTM) models: mean average error (MAE) by number of history encounters, stratified by child sex.

Table 3 and Figure 2 show the results of the LTSM models. Models with five or eight history encounters were determined to more accurately predict the patient’s BMI than models using two or three history encounters. These models fit the observed data well, as shown by the mean average error and correlation between actual BMI and predicted BMI. Models were not trained with more than eight encounters due to concerns of reduced data quantity. Mean average error and correlation estimates were less optimal when using two or three history encounters, with the highest mean average error (1.49 for boys and 1.60 for girls) and the lowest correlation between actual and predicted BMI observed using two history encounters (R^2^ = 0.55 in the boy only model and R^2^ = 0.49 in the girl only model). Moreover, the K-fold standard deviation was low for both the mean average error and the R^2^ in models with five and eight history encounters, indicating that these models were not susceptible to the selection of the training data and were more likely to generalize to new data. We observed higher K-fold standard deviations in models with two or three history encounters, suggesting less optimal performance in predicting BMI.

The above-mentioned advantages of the five and eight history encounter models were achieved despite having longer prediction horizons compared to the two or three history encounters models. For instance, the five history encounters boy model had an average prediction horizon of more than 20 weeks. That is, the model predicted BMI, on average, 20 weeks into the future. Conversely, the two history encounters model had an average prediction horizon of less than 18 weeks.

We did not observe significant model differences between boys and girls. The combined model showed optimal performance with the lowest mean average error (0.98, SD = 0.03) and the highest correlation (R^2^ = 0.72), likely owing to the greater number of patients included.

Within the entire cohort, the mean age at which children reached five clinical encounters was 10.1 months with a standard deviation of 6.5 months.

## 4. Discussion

The purpose of this study was to understand the importance of historical health data in developing machine learning models to identify pediatric patients with increased risk of future overweight and obesity. Our LSTM models suggest that clinical data from at least five clinical encounters are needed to accurately predict child BMI prior to age four years with prediction horizons approximately 20 weeks in the future. In contrast to prior research [39], our combined model performed better than the models separated by sex, negating the need to develop and employ separate models for boys and girls.

Although previous studies have successfully applied machine learning to predict childhood obesity [14], few have investigated the application of these models in clinical care [28]. Our model could be employed in a pediatric clinical setting to dynamically track and predict children’s BMI progression, facilitating obesity prevention through anticipatory guidance during each wellness visit. The results also suggest that having height and weight data from at least five clinical encounters may be necessary to accurately predict future BMI values. Encouragingly, the majority of patients in our sample achieved this threshold within the first 17 months of life, with 10 months being the average age at which children reached five clinical encounters. This suggests that employing our model to identify children at risk for suboptimal weight outcomes is feasible in very early childhood.

The input variables used by our model are consistent with previous findings in the literature [13]. For instance, characteristics of children’s sleep such as duration, timing, and quality have been associated with obesity [41,42]. In this study, we conducted an ablation test on the two sleep quality variables (i.e., frequency of nighttime waking and parental perception of sleep quality) for the combined boys and girls model with five history encounters. The result of the ablation test shows a higher mean average error (1.03 vs. 0.98) with a larger standard deviation (0.07 vs. 0.03). The BMI correlation also dropped from 0.72 to 0.70, underscoring the important association of early sleep quality for the prediction of children’s obesity risk.

Pediatricians are well-positioned to provide parents with information regarding obesity risk in early life, but many consensus guidelines recommend obesity screening in the pediatric setting only after 2 years of age when the “tipping point” of obesity onset may have already passed [43]. Further, meta-analyses indicate that BMI surveillance and counseling have only marginal effects on reducing children’s BMI [44]. There is evidence that unhealthy weight gain in very early childhood of age tracks into later childhood, adolescence, and adulthood [10,11], which suggests that new approaches to help providers and parents address this problem are needed. Our screener, administered in the clinic setting, could help identify very young children at risk of unhealthy weight gain, enabling preventive counseling focused on healthy feeding, activity, and family lifestyle behaviors. Even though our findings show statistical support for postponing BMI prediction until it is possible to obtain information from five clinical encounters, the proposed models still facilitate early identification and intervention as existing guidelines recommend at least this many pediatric visits by six months of age [18]. The prediction horizon of 20 weeks and the frequency of encounters during children’s first year of life means that there are numerous opportunities for providers to monitor growth, identify weight issues, and take appropriate action.

Consistent with prior research [45], the performance of our models diminished as the temporal distance between the acquisition of the exposure variables and the time of BMI prediction in the future increased. While requiring only two history encounters is attractive in practice because it enables the use of the model for a wider population, the high mean average error of the resulting predictive models makes their utility to predict obesity risk limited. The model’s improvement when using five history encounters suggests that more clinical data are needed before one can correctly predict future BMI. However, further research is needed to evaluate the reproducibility and generalizability of our models before they can be applied in clinical practice for similar and related populations. Future work may wish to investigate the relative importance of the variables in our model using an external validation dataset and by conducting ablation experiments as performed in the present study for the subjective sleep quality variables.

Machine learning has been widely applied in the field of obesity research, both for the prediction of future weight outcomes and for identifying targets for intervention. Several previous studies proposed classifiers for obesity in both adults and for early childhood. For instance, Thamrin et al. [46] used linear regression and various machine learning approaches (Bayesian networks and CART models) to classify adults 18 and older as having or not having obesity based on survey data on indicators such as age, parental obesity, and activity level. Here, we predict children’s future BMI rather than classify risks for obesity. We stipulate that the transparency of our proposed approach can better support intervention. Another earlier study by Dugan et al. [47] used longitudinal data from CHICA to compare different machine learning techniques (decision trees, random forest, and Bayesian networks) using 167 features from the first 2 years of life. They found that decision trees provided the best accuracy when predicting obesity between ages 2 and 10 years. Our study expands on this work by using historical data to predict children at risk for obesity. Other research focused on machine learning and obesity prediction has provided thresholds for obesity rather than BMI [48–50], which may not be as applicable for patients at younger ages. The models proposed in the present paper estimate exact BMI values and are dynamic. They predict future BMI based on the nearest history and can therefore be used for children of varying ages. Moreover, the proposed models leverage routinely-collected EHR data, which is a practical approach compared with previous models that, for example, predict obesity using more costly and less accessible genetic data [48,51]. Importantly, the limited number of features we identify makes our model practical for use in other settings. Although the relatively narrow set of variables we identify are not all typically included in the EHR, they could be easily collected using existing screeners [28]. This data collection approach was successfully used in previous studies to obtain child birthweight and weight change between birth and 6, 9, and 12 months [52]; and to obtain data on paternal weight, maternal smoking, and breastfeeding [53].

Our study is subject to some limitations. First, it is possible that our results may be confounded by child age. While the distribution of the data (Table 2) shows that the average at encounter is approximately 68 weeks for all cohorts, patients with five or eight encounters may be older than those with two or three encounters. Their BMI may be more stable and easier to predict. This potential for confounding is the subject of a current investigation. In addition, the EHR data within the OPEL database is derived from a predominately low-income, urban population in Indianapolis, IN. Additional work in other populations is needed to externally validate our findings, as children’s growth patterns may vary by socioeconomic factors [54]. Finally, we were unable to examine other variables that are potentially impactful to children’s early weight gain, like physical activity, as they were not included in the OPEL database. Future research may wish to incorporate such measures for a better understanding of the children’s weight trajectories.

## 5. Conclusions

The present study shows that five history encounters and a limited number of exposure variables are sufficient to predict BMI for both boys and girls in very early childhood. These findings can inform efforts to identify infants at risk of developing overweight and obesity. We envision using the proposed model in a pediatric clinic to dynamically track the progression of children’s BMI four months into the future during each wellness visit. Our findings have implications for future work aimed at early identification and intervention of obesity, as well as for other chronic diseases that begin in early life.

## Figures and Tables

**Table 1.** Features from the OPEL database used in the analysis.

Category	Source	Description
Prenatal	MCPHD	Maternal risk factor during pregnancy
	MCPHD	Method of delivery: vaginal versus cesarean section
	MCPHD	Child’s birthweight (in grams)
Demographic	MCPHD	Child sex
	CHICA	Child’s ethnicity
	CHICA	Child’s age at the clinical encounter
	CHICA	Preferred language of the child
	MCPHD	Biological mother’s age at delivery
	MCPHD	Biological mother’s race and ethnicity
	MCPHD	Father’s race and ethnicity
Environmental	CHICA	Blood lead level
	CHICA/MCPHD	Flag for if the child has ever been enrolled in the WIC program
	SAVI	Percentage of the local population living in a food desert, based on child’s address at birth
	CHICA	Parent is confident filling out health forms
	CHICA	Who attended the visit (e.g., mother, father, grandparent, etc.)
	CHICA	Flag for low health literacy risk, as determined by a validated screener
	CHICA	Parent response to “Are all the doors in your house that lead outside, to stairs, or potentially dangerous areas secured against [child] opening them?”
Developmental	CHICA	Flag for developmental delay
	CHICA	Parent reports concerns about the child’s behavioral development
Sleep Quality	CHICA	Parent response to “Does [child] often wake up one or more times per night, and does an adult go to him/her?”
	CHICA	Parent response to “Do you think [child] has a sleep problem?”
Clinical	CHICA	Type of clinic visit (routine versus sick visit)
	CHICA	Prior BMI measurements
	CHICA	Time between clinical encounters

**Table 2.** Number of patients, average BMI, age, and number of encounters per patients included in the training and testing datasets.

Population	N	BMI	Age at Encounter (Weeks)	Encounters per Patient*
			Mean (SD)	
Training Cohort				
Male	2694	16.79 (2.26)	67.54 (57.43)	12.56 (4.44)
Female	2614	16.39 (2.22)	66.75 (57.22)	12.01 (3.69)
Combined	5308	16.59 (2.25)	67.16 (57.33)	12.29 (4.10)
Testing Cohort				
Male	657	16.71 (2.20)	69.07 (58.09)	12.55 (4.18)
Female	649	16.38 (2.20)	67.28 (56.92)	12.28 (4.17)
Combined	1306	16.55 (2.21)	68.19 (57.52)	12.42 (4.18)

SD, standard deviation;

* Represents the average number of encounters during the timeframe of analysis.

**Table 3.** Results from the long short-term memory (LSTM) models: mean average error, Pearson’s correlation coefficient, and mean prediction horizon in weeks.

History (Encounters)	MAE (SD)	R^2^	Prediction Horizon (Weeks)
Boy Cohort				
8	1.04 (0.06)	0.68 (0.02)	21.56 (17.06)
5	1.02 (0.04)	0.68 (0.02)	20.48 (16.87)
3	1.37 (0.21)	0.58 (0.07)	18.83 (16.1)
2	1.49 (0.36)	0.55 (0.09)	17.79 (15.73)
Girl Cohort				
8	1.03 (0.03)	0.71 (0.01)	22.71 (17.39)
5	1.06 (0.04)	0.69 (0.01)	21.18 (17.22)
3	1.35 (0.18)	0.62 (0.04)	19.36 (16.37)
2	1.60 (0.45)	0.49 (0.14)	18.25 (16.02)
Combined Cohort				
5	0.98 (0.03)	0.72 (0.01)	20.87 (17.09)

Each entry is the mean value of all folds in a 5 K-fold evaluation. MAE, mean average error; SD, standard deviation.

## Data Availability

The data presented in this study are available on request from the corresponding author. The data are not publicly available due to privacy laws.

## Appendix A Complete list of starting features before LASSO reduction by data source.

**Table:**

Name	Description	Data Source
weight	child’s weight at visit	CHICA
wtcentile	child’s weight percentile	CHICA
height	child’s height at visit	CHICA
htcentile	child’s height percentile	CHICA
insurance	What kind of insurance, if any, the patient has at time of visit	CHICA
any_household_members_smoke	Do any of the people that live with the child smoke?	CHICA
car_seat_position_01	Does the child use a car seat, and if so, which way is it facing?	CHICA
fluoride_supplemented	Does the child have fluoride supplemented somehow through consumption?	CHICA
has_smoke_detector	Does the child’s living area have a smoke detector?	CHICA
hc	child’s head circumference in centimeters	CHICA
hccentile	child’s head circumference percentile	CHICA
know_how_to_save_choking_child	Do the child’s caregivers know how to perform the Heimlich maneuver on a choking child?	CHICA
left_alone_in_water	Is the child left alone in water?	CHICA
lg_failed	What question of the language developmental test did the child fail on?	CHICA
maternal_depression_concern	Based on a questionnaire, is there a concern that the mom might be depressed?	CHICA
medicationallergies	Does the child have any medication allergies and have the allergies been confirmed by a doctor or only reported by the family?	CHICA
painqualitative	Is the child in pain, yes, no or NA?	CHICA
ps_passed	What is the highest passed question for the psychosocial developmental test?	CHICA
sleeps_on_side_or_back	Does the child sleep on their side or back?	CHICA
slept_on_stomach_ever	Does the child ever sleep on their stomach?	CHICA
uses_walker	Does the child use a walker?	CHICA
baby_left_alone_could_fall	Is the baby ever left alone where they could fall?	CHICA
sleeps_unsafe_soft_surface	Does the child sleep on an unsafe soft surface such as a mattress that they can suffocate on if they sleep facedown?	CHICA
tested_smoke_detector	If the child’s living place has a smoke detector, has it been tested as working?	CHICA
abdomen_exam	If the child’s abdomen is examined, is it abnormal or normal?	CHICA
back_exam	If the child’s back is examined, is it abnormal or normal?	CHICA
chestlungs_exam	If the child’s chest or lungs are examined, is it abnormal or normal?	CHICA
extgenitalia_exam	If the child’s external genitalia is examined, is it normal or abnormal?	CHICA
extremities_exam	If the child’s extremities (hands, feet, nose, ears) are examined, are they normal or abnormal?	CHICA
fm_passed	For the fine motor skills developmental test, what is the highest question passed?	CHICA
general_exam	If the child had a general exam, was it normal or abnormal?	CHICA
gm_passed	For the gross motor skills developmental test, what is the highest passed question?	CHICA
head_exam	If the child’s head is examined, is it normal or abnormal?	CHICA
heartpulses_exam	If the child’s heart and pulse are examined, is it normal or abnormal?	CHICA
lg_passed	For the language developmental test, what is the highest scoring passed question?	CHICA
neuro_exam	If a neurological battery is done, was it normal or abnormal?	CHICA
nodes_exam	If the lymph nodes are checked, were they normal or abnormal?	CHICA
nosethroat_exam	If the nose and throat are examined, are they normal or abnormal?	CHICA
skin_exam	If the child’s skin is examined, was it normal or abnormal?	CHICA
teethgums_exam	If the child’s teeth and gums are examined, were they normal or abnormal?	CHICA
preferred_language	Does the child have a preferred language and if so, is it English or Spanish?	CHICA
burns_knowledge	Does the caregiver have knowledge of how to take care of burns?	CHICA
firearms_at_home	Are there any firearms in the home?	CHICA
firearms_where_visits	Are there any firearms where the visit is taking place?	CHICA
has_stairway_gates	Are there child safety gates over the stairways?	CHICA
household_products_out_of_reach	Are household cleaning products such as bleach out of the reach of children?	CHICA
matches_lighters_safe	Are matches and lighters kept in a safe manner? childproof wheel, out of reach, etc.	CHICA
play_area_fenced	Is the child’s play area fenced in?	CHICA
pool_at_house	Is there a pool the child can access?	CHICA
chica_devscreen_status	This is a developmental screening that states whether the child is developing normally or if they are developmentally delayed and indicate which developmental screenings have been done.	CHICA
seen_dentist	Has the child ever been seen by a dentist? This is unlikely to be true until after the child has teeth.	CHICA
taking_medications	Is the child on any medications and if so, has this list of medications been confirmed to be accurate?	CHICA
tv_in_room	Is there a TV in the child’s bedroom?	CHICA
tv_over_2hrs	Does the child watch TV for more than two hours every day?	CHICA
uses_bottle	Does the child use a bottle to eat?	CHICA
asthmastatus	Does the child have any asthma symptoms and if so, are they persistent, intermittent, uncontrolled/controlled?	CHICA
chica_devscreen_sx	Are there any developmental concerns?	CHICA
lye_drain_cleaners_in_house	Are there any lye, drain, or other more dangerous cleaners in the house?	CHICA
ps_failed	What question of the psychosocial test did the child fail on?	CHICA
stop_at_curb	Does the child stop at curbs or run straight without stopping?	CHICA
wears_bike_helmet	Does the child wear a bike helmet for activities where one is recommended?	CHICA
insurancename	What kind of insurance does the child have?	CHICA
parents_confident_filling_out_	Do the parents appear confident filling out forms?	CHICA
parents_need_help_reading	Do the parents need help reading forms?	CHICA
ten_childrens_books_in_home	Are there at least 10 children’s books in the home available to the child?	CHICA
visittype	Is this a visit because the child is sick?	CHICA
chica_adhd_sx	Is the child having symptoms of ADHD?	CHICA
constipation_sx	Is the child having symptoms of constipation?	CHICA
firearms_kept_unloaded	Are any firearms kept unloaded in the household?	CHICA
look_both_ways	Does the child look both ways before crossing the street?	CHICA
unsupervised_near_water	Is the child left unsupervised near water?	CHICA
firearms_discussed	Has firearm safety been discussed with the child?	CHICA
grades_dropped_lately	Has the child’s school grades dropped recently?	CHICA
knows_how_to_swim	Does the child know how to swim?	CHICA
rides_bike_in_street	Does the child ride their bike in the street?	CHICA
school_suspension_this_year	Has the child been suspended from school this year?	CHICA
snoring	Have parents noticed that the child snores?	CHICA
special_education_classes	Does the child attend special education classes?	CHICA
escape_plan_for_fire	Has the family discussed a house fire escape plan with their child? Older children version of smoke_alarm_knows_what_to_do	CHICA
informant	What household member is answering the questions?	CHICA
smoke_alarm_knows_what_to_do	Does the child know what to do when the smoke/fire alarm is triggered? Younger children version of escape_plan_for_fire	CHICA
specialneeds	Does the child have special needs or accomodations? Such as ear defenders, speech therapist, etc…	CHICA
visit_attendee	What household member is attending the visit but not necessarily the informant?	CHICA
hot_water_heater_adjusted	Has the water heater been adjusted so the water can only be heated to 120 degrees farenheit? This is a scalding concern.	CHICA
plastic_wrappers_secured	Are plastic wrappers in the environment secured or left in an accessible area? This is a suffocation hazard.	CHICA
taking_solid_food	Is the child eating solid food yet?	CHICA
cutting_food_bite_size	Are the child’s solid foods being cut into bite size pieces before being given to the child? If no, this is a choking/suffocation hazard.	CHICA
carries_hot_liquids	Is the child allowed to carry hot liquids? This is a burn hazard.	CHICA
play_area_cooking	Does the child have an area to play and be safely in away from cooking area while caregiver is cooking? This is a burn risk if not.	CHICA
safety_latches_installed	Have safety latches been installed in the house?	CHICA
car_seat_inspection	Has the child’s car seat been inspected and if so, is it forward or rear facing? Rear facing is the safer option.	CHICA
developmental_referral	Has the child been referred to developmental testing and if so, have only the first steps been taken or has the appointment been made?	CHICA
fm_failed	What difficulty of the fine motor skills test did the child fail on?	CHICA
correctedvision	Does the child wear glasses or contact lenses?	CHICA
firearms_friends	Does the child go to friend’s houses which have firearms?	CHICA
plays_dangerous_items	Does the child play with dangerous items?	CHICA
wears_sports_protective_gear	Does the child wear protective gear while playing sports?	CHICA
safety_caps_on_bottles	Are there child safety caps on pill bottles around the child?	CHICA
wears_life_jacket	Does the child wear a life jacket in situations where that is recommended?	CHICA
bedtime_media	Does the child use media products at bedtime?	CHICA
daytime_sleepiness	Is the child sleepy during the day?	CHICA
questionnaireinformants	Which caregiver filled out the questionnaire?	CHICA
sleep_quantity	Does the child get sufficient or insufficient sleep?	CHICA
chica_t2dm_fh	Does the child’s medical records include family history?	CHICA
chica_t2dm_gdm	Did the child’s mother have gestational diabetes?	CHICA
chica_t2dm_lga	Was the child large for their gestational age during pregnancy?	CHICA
epilepsy_history	Is there a family-reported family history of epilepsy?	CHICA
breast_feeding_help_needed	Does the mother need help breastfeeding?	CHICA
oral_exam	Has the child’s mouth been examined and if so, was it normal or abnormal?	CHICA
bp_eval	Has the child’s blood pressure been evaluated and if so, was it elevated once or repeatedly elevated? There was no option for hypotensive in this variable.	CHICA
empty_container_after_use	Do caregivers empty bathwater container immediately after use? This is a drowning risk if no.	CHICA
well_water	Does the child’s household run off well-water? Well-water is a contamination concern.	CHICA
lowliteracyrisk	Is the child at risk of low literacy and if so, have they gone to a clinic to help?	CHICA
morning_headaches	Does the child have headaches in the morning or wake up with a headache?	CHICA
nocturnal_enuresis	Does the child wet the bed/pee during sleep? This question is for kids who are out of diapers.	CHICA
stops_breathing_at_night	Does the child’s caregiver know if the child stops breathing during the night?	CHICA
trouble_breathing_at_night	Does the child’s caregiver know if the child has trouble breathing during the night?	CHICA
wakes_with_snort	Does the child caregiver know if the child wakes up with a snort?	CHICA
rides_after_dark	Does the child ride the bike after sunset?	CHICA
knows_rules_of_road	Does the child know traffic rules?	CHICA
swims_fast_moving_water	Does the child swim in fast-moving water such as a river?	CHICA
chica_adhd_dx	Does the child have an ADHD diagnosis?	CHICA
doors_secure	Are the doors in the child’s home secure?	CHICA
sharp_edged_furniture	Are there sharp-edged furniture in the child’s home?	CHICA
pulseox	What was the child’s pulse oxygenation percentage at visit?	CHICA
has_window_guards	Does the child home have window guards?	CHICA
play_equipment_protected	Does the child play on safe playground equipment?	CHICA
asthmasymptoms	Does the child have symptoms of asthma?	CHICA
gm_failed	What gross motor test did the child fail on?	CHICA
chica_adhd_side_effects	Does the child experience side effects from their ADHD medication?	CHICA
irondeficiencyscreenqualitativ	Has the child been checked for iron deficiency and if so, what were the results?	CHICA
chica_devscreen_management	Is the child part of activities specifically made for children?	CHICA
normal_newborn_screen	Did the child have the normal newborn screen and if so, what were the results?	CHICA
vaccine_given	Has the child had the HPV, Tdap, or meningococcal vaccine given?	CHICA
anhedonia_past_few_weeks	Has the child been anhedonic/apathetic the last few weeks?	CHICA
cigarettes_snuff_friend	Does the child’s friend or friend’s household use cigarettes or snuff?	CHICA
cigarettes_snuff_live_with	Does someone the child lives with use snuff?	CHICA
ever_use_tobacco	Has the child ever used tobacco?	CHICA
has_drunk_alcohol	Has the child drunk alcohol at all?	CHICA
has_gotten_high	Has the child used an illicit substance?	CHICA
has_had_forced_sex_act	Has the child experienced a forced sex act?	CHICA
has_had_intercourse	Has the child had intercourse?	CHICA
sad_past_few_weeks	Has the child been sad in the past few weeks?	CHICA
suicide_concerns	Is there a concern of suicidality for the child?	CHICA
used_marijuana	Has the child used marijuana?	CHICA
interested_birth_control	Is the child interested in contraception?	CHICA
ready_to_quit	Is the child ready to quit smoking cigarettes?	CHICA
watches_tv	Does the child watch TV?	CHICA
sleep_problems	Does the child have problems sleeping?	CHICA
nobp	Child did not cooperate in visit; Could not check blood pressure.	CHICA
nohearing	Child did not cooperate in visit; Could not perform hearing exam.	CHICA
risk_based_hearing_screen	Has the child undergone a hearing screen that was ordered based on high risk?	CHICA
chica_devscreen_treatment	Does the child have a written care plan or access to family support services?	CHICA
anxiety_status	Does the child have an anxiety diagnosis, or has this questionnaire been deferred?	CHICA
phq9_score	What was the mother’s depression score on the phq9?	CHICA
driven_with_drunk	Has the child driven while drunk?	CHICA
drunk_and_activity	Has the child been drunk while doing an activity?	CHICA
drunk_last_month	Has the child been drunk in the last month?	CHICA
family_substance_abuse	Does the child’s family abuse any substances?	CHICA
happy_how_things_going	Is the child happy with life?	CHICA
uses_drugs	Does the child use drugs?	CHICA
sudep_risk_counseling	Is the child at risk for sudden unexpected death from epilepsy? If so, is the risk high or low?	CHICA
surgical_hx	Has the child had their tonsils and adenoids removed?	CHICA
feed_at_night	Does the child eat at night?	CHICA
contraceptive_method_discussed	Has birth control been discussed with the child such as condoms and hormonal birth control?	CHICA
abuse_otc	Does the child abuse over the counter medication?	CHICA
abuse_steroids	Does the child abuse steroids drugs?	CHICA
criticized_for_drinking	Has the child been criticized for drinking?	CHICA
friends_use_drugs	Has the child’s friends used drugs (other than alcohol/caffeine) in the last month?	CHICA
friend_drunk_last_month	Has the child’s friends been drunk in the last month?	CHICA
fun_in_past_two_weeks	Does the child think they’ve had fun in the last two weeks?	CHICA
bike_has_coaster_brakes	Does the child’s bike have coaster brakes? Coaster brakes allow you to pedal backwards to brake.	CHICA
past_depression_or_suicide	Has the child had any previous history of depression or suicidality?	CHICA
immune_compromise	Is the child immuno-compromised?	CHICA
prescription_for_cessation	Is the child on a prescribed nicotine replacement drug?	CHICA
intercourse_past_year	Has the child had intercourse in the last year?	CHICA
might_be_pregnant	Could the child be pregnant?	CHICA
medication	Does the child have a Ritalin prescription?	CHICA
depression_workup	Is there a developed safety plan for the child’s depression?	CHICA
chica_autism_risk	Is the child at a higher risk of autism due to family history?	CHICA
tooth_erupted	Has the child had a tooth erupt from beneath the gums yet?	CHICA
autism_behavior_problems	Does the child have autism related behavior problems?	CHICA
autism_cam	Does the child use complementary alternative medicine for autism?	CHICA
autism_financial_concerns	Are there financial concerns related to the child’s autism such as paying for therapy?	CHICA
autism_parent_needs_respite	Is the child’s caregiver in need of a break? i.e., showing symptoms of caregiver burnout	CHICA
patient_in_mental_health	Is the child undergoing mental health care?	CHICA
food_insecurity	Is the child’s caregiver worried about getting enough food and if so, has this been MD confirmed or resolved?	CHICA
rental_status	Is the child’s rental home clean & safe vs having issues, and has this been confirmed by an MD?	CHICA
snapdeniedlast30days	Has the child’s SNAP(food stamps) been denied in the last 30 days?	CHICA
utility_status	Has the child’s household had one of their utilities (water, power, heat, gas) shut off? Yes, no, or yes but not heat.	CHICA
mlp_condition_type	Is the child’s family going through an eviction, on the SNAP program, or renting?	CHICA
wakes_up_one_or_more_times_a_n	Does the child wake up at least once during the night?	CHICA
wakes_up_and_needs_help_to_sleep	Does the child wake up at night and need help getting back to sleep?	CHICA
sleeps_on_back	Does the child sleep on their back?	CHICA
slept_on_stomach_side_ever	Does the child ever sleep on their stomach or side?	CHICA
abuse_concern	Is there a concern that the child is being abused?	CHICA
constipation_dx	Has the child been diagnosed with constipation?	CHICA
parent_thinks_child_has_sleep_pr	Do the caregivers think that the child has problems with their sleep?	CHICA
eyesvision_exam	Did the child have a normal or abnormal vision exam?	CHICA
breastfed	Is the child being breastfed at this time?	CHICA
psfsicklecell	Result of pre-screening form on tablet for sickle cell anemia.	CHICA
negativeenvironmentalhistory	Was the child potentially exposed to something negative in their environment such as tuberculosis or lead?	CHICA
negativenutritionhistory	Did the child have nutrition problems such as early introduction to cow milk or needing low iron formula?	CHICA
negativepastmedicalhistory	Did the child have a low birth weight?	CHICA
cholesterol_screen	Is the child at risk of high cholesterol based on parental history?	CHICA
earshearing_exam	Did the child have a normal or abnormal hearing exam?	CHICA
hearingleft	Does the child have full or partial hearing in their left ear?	CHICA
hearingright	Does the child have full or partial hearing in their right ear?	CHICA
ppd_result	What was the result of the mother’s post-partum depression assessment?	CHICA
venousbloodleadqualitative	How much lead was in the child’s blood, if tested?	CHICA
mother_bmi	Maternal body mass index	MCPHD
PNC_Clinic_Type	Type of prenatal care clinic	MCPHD
Sex	Child’s sex	MCPHD
FATHER_OCCUP_DSCRP	Is child’s father employed at time of birth?	MCPHD
MomNativeAm	Is child’s mother Native American?	MCPHD
Mother_Weight_Gain_P	How many pounds the mother has gained during pregnancy.	MCPHD
MARRIED_NOW	Are child’s parents married at time of birth?	MCPHD
APGAR5	Appearance, Pulse, Grimace, Activity, and Respiration at five minutes post birth. Score of 10 is good; one is bad.	MCPHD
BIRTH_WEIGHT_GRAM	Birth weight in grams from modern birth certificate	MCPHD
finalroute	How was the child delivered?	MCPHD
HEP_B_TEST	Was hepatitis B vaccine given at birth?	MCPHD
Apgar1	Appearance, Pulse, Grimace, Activity, and Respiration at 1 min post birth. Score of 10 is good; one is bad.	MCPHD
Dad_Race9Eth	race of child’s father	MCPHD
Mom_Race9Eth	race of child’s mother	MCPHD
PREN_VISIT_NBR	number of prenatal care visits	MCPHD
EST_GEST	estimated gestation in weeks	MCPHD
MOTHER_AGE	age of the mother at birth in years	MCPHD
FATHER_AGE	age of the father at birth in years	MCPHD
PREVIOUS_LIVE_NBR	How many living babies has the mother giving birth to before?	MCPHD
plurality	Is this a plural or singleton birth? (twins)	MCPHD
BREAST_FED	Was the child breast-fed at hospital release?	MCPHD
MOTHER_ED	mother’s education level in years	MCPHD
FATHER_ED	father’s education level in years	MCPHD
LD_MECONIUM	delivery complication: was there meconium present at delivery?	MCPHD
LD_NONE	no delivery complications	MCPHD
LD_NON_VERTEX	delivery complication: child in non- vertex position	MCPHD
firstpnc	prenatal care initiated in first trimester	MCPHD
wtgrams	child’s birth weight in grams	MCPHD
PREV_BIRTH_TOTAL	number of previous live births—all birth certificates	MCPHD
Kotelchuck	adequacy of prenatal care index	MCPHD
mdpsmoke	Did the mother smoke during pregnancy?	MCPHD
abcond	Were abnormal conditions present at birth?	MCPHD
anomaly	Was a congenital anomaly found?	MCPHD
infect	maternal infections	MCPHD
labdel	labor and delivery	MCPHD
mmorb	maternal morbidity	MCPHD
methdel	method of delivery	MCPHD
oblab	obstetrical labor	MCPHD
obproc	obstetrical procedures	MCPHD
risk	maternal risk factor	MCPHD
RACE	race of the child	CHICA
ETHN	ethnicity of the child	CHICA
wic_ever	Has the child ever been in the WIC program?	CHICA/MCPHD
PERINPOVN1	persons living in poverty as percentage of population	SAVI
VIOLENTN2	violent crime (including simple assaults) per 1000 people	SAVI
VIOLNSTN2	violent crime (not including simple assaults) per 1000 people	SAVI
AGGVASLTN2	aggravated assaults per 1000 people	SAVI
ROBBERYN2	robberies per 1000 people	SAVI
PROPERTYN2	property crime per 1000 people	SAVI
THFTVHN2	vehicle thefts per 1000 people	SAVI
BURGLARYN2	burglaries per 1000 people	SAVI
WALKSCORE	walkability score	SAVI
FRRDTRAN1	free and reduced lunch program participants as percentage of enrollment	SAVI
POVB185N1	population below 185% poverty (proxy for reduced lunch)	SAVI
POVB125N1	population below 125% poverty (proxy for free lunch)	SAVI
RESNEWPEN1	total residential building permits per 100 housing units	SAVI
COMMALLPN1	total commercial building permits per 100 housing units	SAVI
TREE_CANOPY	tree canopy as percentage of land area	SAVI
PCT_POP_FOOD_DESERT	percentage of population far from grocery stores	SAVI

## References

[1] Friedrich M Global obesity epidemic worsening. JAMA 2017, 318, 603. 10.1001/jama.2017.10693 28810033

[2] GBD 2015 Obesity Collaborators. Health effects of overweight and obesity in 195 countries over 25 years. N. Engl. J. Med 2017, 377, 13–27. 28604169 10.1056/NEJMoa1614362 PMC5477817

[3] Freedman DS; Khan LK; Dietz WH; Srinivasan SR; Berenson GS Relationship of childhood obesity to coronary heart disease risk factors in adulthood: The Bogalusa Heart Study. Pediatrics 2001, 108, 712–718. 11533341 10.1542/peds.108.3.712

[4] Must A; Strauss RS Risks and consequences of childhood and adolescent obesity. Int. J. Obes. Relat. Metab. Disord 1999, 23 (Suppl. 2), S2–S11. 10.1038/sj.ijo.0800852 10340798

[5] Dietz WH Overweight and precursors of type 2 diabetes mellitus in children and adolescents. J. Pediatr 2001, 138, 453–454. 11295702 10.1067/mpd.2001.113635

[6] Taveras EM; Rifas-Shiman SL; Camargo CA Jr.; Gold DR; Litonjua AA; Oken E; Weiss ST; Gillman MW Higher adiposity in infancy associated with recurrent wheeze in a prospective cohort of children. J. Allergy Clin. Immunol 2008, 121, 1161–1166.e3. 18466784 10.1016/j.jaci.2008.03.021 PMC3253368

[7] Dietz WH Childhood weight affects adult morbidity and mortality. J. Nutr 1998, 128 (Suppl. 2), 411S–414S. 9478038 10.1093/jn/128.2.411S

[8] World Health Organization. Commission on the Social Determinants of Health; WHO: Geneva, Switzerland, 2008.

[9] General Assembly of the United Nations. High-Level Meeting on Non-Communicable Diseases. 2011. Available online: http://www.un.org/en/ga/president/65/issues/ncdiseases.shtml (accessed on 1 June 2021).

[10] Li H; Stein AD; Barnhart HX; Ramakrishnan U; Martorell R Associations between prenatal and postnatal growth and adult body size and composition. Am. J. Clin. Nutr 2003, 77, 1498–1505. 12791630 10.1093/ajcn/77.6.1498

[11] Rogers I The influence of birthweight and intrauterine environment on adiposity and fat distribution in later life. Int. J. Obes 2003, 27, 755–777. 10.1038/sj.ijo.0802316 12821960

[12] Barlow SE; Expert C Expert committee recommendations regarding the prevention, assessment, and treatment of child and adolescent overweight and obesity: Summary report. Pediatrics 2007, 120 (Suppl. 4), S164–S192. 18055651 10.1542/peds.2007-2329C

[13] Baidal JAW; Locks LM; Cheng ER; Blake-Lamb TL; Perkins ME; Taveras EM Risk factors for childhood obesity in the first 1000 days: A systematic review. Am. J. Prev. Med 2016, 50, 761–779. 26916261 10.1016/j.amepre.2015.11.012

[14] LeCroy MN; Kim RS; Stevens J; Hanna DB; Isasi CR Identifying Key Determinants of Childhood Obesity: A Narrative Review of Machine Learning Studies. Child. Obes 2021, 17, 153–159. 33661719 10.1089/chi.2020.0324 PMC8418446

[15] Wiemken TL; Kelley RR Machine Learning in Epidemiology and Health Outcomes Research. Annu. Rev. Public Health 2019, 41, 21–36. 31577910 10.1146/annurev-publhealth-040119-094437

[16] Zhang S; Tjortjis C; Zeng X; Qiao H; Buchan I; Keane J Comparing data mining methods with logistic regression in childhood obesity prediction. Inf. Syst. Front 2009, 11, 449–460.

[17] Beam AL; Kohane IS Big data and machine learning in health care. JAMA 2018, 319, 1317–1318. 29532063 10.1001/jama.2017.18391

[18] Simon GR; Baker C; Barden GA 3rd; Brown OW; Hardin A; Lessin HR; Meade K; Moore S; Rodgers CT; Hammer LD; 2014 Recommendations for Pediatric Preventive Health Care. Pediatrics 2014, 133, 568–570. 24567012 10.1542/peds.2013-4096

[19] Wolf ER; Hochheimer CJ; Sabo RT; DeVoe J; Wasserman R; Geissal E; Opel DJ; Warren N; Puro J; O’Neil J; Gaps in well-child care attendance among primary care clinics serving low-income families. Pediatrics 2018, 142, e20174019. 30305388 10.1542/peds.2017-4019 PMC7063686

[20] Siddiqui H; Rattani A; Woods NK; Cure L; Lewis R; Twomey J; Smith-Campbell B; Hill TJ A Survey on Machine and Deep Learning Models for Childhood and Adolescent Obesity. IEEE Access 2021, 9, 157337–157360.

[21] Grow HM; Cook AJ; Arterburn DE; Saelens BE; Drewnowski A; Lozano P Child obesity associated with social disadvantage of children’s neighborhoods. Soc. Sci. Med 2010, 71, 584–591. 20541306 10.1016/j.socscimed.2010.04.018 PMC2928553

[22] Fiechtner L; Block J; Duncan DT; Gillman MW; Gortmaker SL; Melly SJ; Rifas-Shiman SL; Taveras EM Proximity to supermarkets associated with higher body mass index among overweight and obese preschool-age children. Prev. Med 2013, 56, 218–221. 23219681 10.1016/j.ypmed.2012.11.023 PMC3837524

[23] Lovasi GS; Schwartz-Soicher O; Quinn JW; Berger DK; Neckerman KM; Jaslow R; Lee KK; Rundle A Neighborhood safety and green space as predictors of obesity among preschool children from low-income families in New York City. Prev. Med 2013, 57, 189–193. 23732240 10.1016/j.ypmed.2013.05.012 PMC3748212

[24] Carroll-Scott A; Gilstad-Hayden K; Rosenthal L; Peters SM; McCaslin C; Joyce R; Ickovics JR Disentangling neighborhood contextual associations with child body mass index, diet, and physical activity: The role of built, socioeconomic, and social environments. Soc. Sci. Med 2013, 95, 106–114. 23642646 10.1016/j.socscimed.2013.04.003 PMC4058500

[25] Papas MA; Alberg AJ; Ewing R; Helzlsouer KJ; Gary TL; Klassen AC The built environment and obesity. Epidemiol. Rev 2007, 29, 129–143. 17533172 10.1093/epirev/mxm009

[26] Dunton GF; Kaplan J; Wolch J; Jerrett M; Reynolds KD Physical environmental correlates of childhood obesity: A systematic review. Obes. Rev. Off. J. Int. Assoc. Study Obes 2009, 10, 393–402. 10.1111/j.1467-789X.2009.00572.x PMC3833101 19389058

[27] Lovasi GS; Hutson MA; Guerra M; Neckerman KM Built environments and obesity in disadvantaged populations. Epidemiol. Rev 2009, 31, 7–20. 19589839 10.1093/epirev/mxp005

[28] Butler ÉM; Derraik JG; Taylor RW; Cutfield WS Prediction models for early childhood obesity: Applicability and existing issues. Horm. Res. Paediatr 2018, 90, 358–367. 30739117 10.1159/000496563

[29] Ziauddeen N; Roderick PJ; Macklon NS; Alwan NA Predicting childhood overweight and obesity using maternal and early life risk factors: A systematic review. Obes. Rev 2018, 19, 302–312. 29266702 10.1111/obr.12640 PMC5805129

[30] Hawkins SS; Oken E; Gillman MW Early in the life course: Time for obesity prevention. In Handbook of Life Course Health Development; Springer: Berlin/Heidelberg, Germany, 2018; pp. 169–196. 31314285

[31] Blake-Lamb TL; Locks LM; Perkins ME; Woo Baidal JA; Cheng ER; Taveras EM Interventions for Childhood Obesity in the First 1000 Days A Systematic Review. Am. J. Prev. Med 2016, 50, 780–789. 26916260 10.1016/j.amepre.2015.11.010 PMC5207495

[32] St. George SM; Agosto Y; Rojas LM; Soares M; Bahamon M; Prado G; Smith JD A developmental cascade perspective of paediatric obesity: A systematic review of preventive interventions from infancy through late adolescence. Obes. Rev 2020, 21, e12939. 31808277 10.1111/obr.12939 PMC6980892

[33] Hochreiter S; Schmidhuber J Long short-term memory. Neural Comput. 1997, 9, 1735–1780. 9377276 10.1162/neco.1997.9.8.1735

[34] Wang Y; Canahuate GM; Van Dijk LV; Mohamed AS; Fuller CD; Zhang X; Marai G-E Predicting late symptoms of head and neck cancer treatment using LSTM and patient reported outcomes. In Proceedings of the 25th International Database Engineering & Applications Symposium, Montreal, QC, Canada, 14-16 July 2021. 10.1145/3472163.3472177 PMC8982996 35392138

[35] Deng Y; Dolog P; Gass J-M; Denecke K Obesity entity extraction from real outpatient records: When learning-based methods meet small imbalanced medical data sets. In Proceedings of the 2019 IEEE 32nd International Symposium on Computer-Based Medical Systems (CBMS), Cordoba, Spain, 5-7 June 2019.

[36] Anand V; Biondich PG; Liu G; Rosenman M; Downs SM Child Health Improvement through Computer Automation: The CHICA system. Stud. Health Technol. Inform 2004, 107, 187–191. 15360800

[37] Bodenhamer DJ; Colbert JT; Comer KF; Kandris SM Developing and sustaining a community information system for central Indiana: SAVI as a case study. In Community Quality-of-Life Indicators: Best Cases V; Springer: Berlin/Heidelberg, Germany, 2011; pp. 21–46.

[38] Kuczmarski RJ; Ogden CL; Grummer-Strawn LM; Flegal KM; Guo SS; Wei R; Mei Z; Curtin LR; Roche AF; Johnson CL CDC growth charts: United States. Adv. Data 2000, 314, 1–27. 11183293

[39] Hammond R; Athanasiadou R; Curado S; Aphinyanaphongs Y; Abrams C; Messito MJ; Gross R; Katzow M; Jay M; Razavian N; Predicting childhood obesity using electronic health records and publicly available data. PLoS ONE 2019, 14, e0215571. 31009509 10.1371/journal.pone.0215571 PMC6476510

[40] Lachenbruch PA; Mickey MR Estimation of error rates in discriminant analysis. Technometrics 1968, 10, 1–11.

[41] Fatima Y; Doi S; Mamun A Sleep quality and obesity in young subjects: A meta-analysis. Obes. Rev 2016, 17, 1154–1166. 27417913 10.1111/obr.12444

[42] Matricciani L; Paquet C; Galland B; Short M; Olds T Children’s sleep and health: A meta-review. Sleep Med. Rev 2019, 46, 136–150. 31121414 10.1016/j.smrv.2019.04.011

[43] Harrington JW; Nguyen VQ; Paulson JF; Garland R; Pasquinelli L; Lewis D Identifying the “tipping point” age for overweight pediatric patients. Clin. Pediatr 2010, 49, 638–643. 10.1177/0009922809359418 20150210

[44] Sim LA; Lebow J; Wang Z; Koball A; Murad MH Brief primary care obesity interventions: A meta-analysis. Pediatrics 2016, 138, e20160149. 27621413 10.1542/peds.2016-0149

[45] Gupta M; Phan T-LT; Bunnell T; Beheshti R Obesity Prediction with EHR Data: A deep learning approach with interpretable elements. arXiv 2019, arXiv:191202655. 10.1145/3506719 PMC9221869 35756858

[46] Thamrin SA; Arsyad DS; Kuswanto H; Lawi A; Nasir S Predicting Obesity in Adults Using Machine Learning Techniques: An analysis of Indonesian Basic Health Research 2018. Front. Nutr 2021, 8, 252. 10.3389/fnut.2021.669155 PMC8255629 34235168

[47] Dugan TM; Mukhopadhyay S; Carroll A; Downs S Machine Learning Techniques for Prediction of Early Childhood Obesity. Appl. Clin. Inform 2015, 6, 506–520. 26448795 10.4338/ACI-2015-03-RA-0036 PMC4586339

[48] Chatterjee A; Gerdes MW; Martinez SG Identification of risk factors associated with obesity and overweight—A machine learning overview. Sensors 2020, 20, 2734. 32403349 10.3390/s20092734 PMC7248873

[49] DeGregory KW; Kuiper P; DeSilvio T; Pleuss JD; Miller R; Roginski JW; Fisher CB; Harness D; Viswanath S; Heymsfield SB; A review of machine learning in obesity. Obes. Rev 2018, 19, 668–685. 29426065 10.1111/obr.12667 PMC8176949

[50] Colmenarejo G Machine Learning Models to Predict Childhood and Adolescent Obesity: A Review. Nutrients 2020, 12, 2466. 32824342 10.3390/nu12082466 PMC7469049

[51] Montañez CAC; Fergus P; Hussain A; Al-Jumeily D; Abdulaimma B; Hind J; Radi N Machine learning approaches for the prediction of obesity using publicly available genetic profiles. In Proceedings of the 2017 International Joint Conference on Neural Networks (IJCNN), Anchorage, AK, USA, 14-19 May 2017.

[52] Santorelli G; Petherick ES; Wright J; Wilson B; Samiei H; Cameron N; Johnson W Developing prediction equations and a mobile phone application to identify infants at risk of obesity. PLoS ONE 2013, 8, e71183. 23940713 10.1371/journal.pone.0071183 PMC3737139

[53] Weng SF; Redsell SA; Nathan D; Swift JA; Yang M; Glazebrook C Estimating overweight risk in childhood from predictors during infancy. Pediatrics 2013, 132, e414–e421. 23858427 10.1542/peds.2012-3858

[54] Vrijkotte TG; Oostvogels AJ; Stronks K; Roseboom TJ; Hof MH Growth patterns from birth to overweight at age 5–6 years of children with various backgrounds in socioeconomic status and country of origin: The ABCD study. Pediatric Obes. 2020, 15, e12635. 10.1111/ijpo.12635 PMC7507194 32237216

